# Notes and News

**Published:** 1891-05-02

**Authors:** 


### Notes and News.

COLLECTED AND COMMUNICATED.

The foundation stone of the new Derbyshire Infirmary will be laid by the Queen on the Thursday in Whit-week. Her Majesty will arrive in Derby at five in the afternoon, and will go first to the Town Hall, where the Mayor will read the Corporation address. The royal party will then proceed to the Infirmary, where a large circular tent, capable of seating fourteen or fifteen hundred persons, will be erected, in the centre of which will be a raised platform, where the actual ceremony of laying the foundation stone will be performed. Admission to this tent will be by ticket, for which a charge of one guinea will be made, the money thus raised being devoted to the Infirmary funds. The ceremony over Her Majesty will be conducted to the Market Place, where the dutiful and loyal addresses of the town and county will be presented.

The annual meeting of the Governors of the Carmarthenshire Infirmary was presided over by Sir James Hill-Jones, K.C.B., V.C. The Secretary read the report for 1890, which showed that the efficiency and condition of the hospital were most satisfactory. The income has shown an increase during the year, but the increase has not been sufficient to warrant the Committee to undertake the repairs which the report described as so necessary. The repairs of the roof and the reflooring of some of the wards cannot be much longer delayed; it is hoped, therefore, that efforts will be made by those who have the welfare of the Infirmary at heart, to obtain such funds as will enable the Committee to undertake this work. After passing the report the meeting elected Mr. E. P. Davies, whose health has compelled him to resign his position as Honorary Surgeon, as consulting Medical Officer to the Institution.

Mr. George Herring, Treasurer of the North-West London Hospital, writing to the Editor, says: "I feel I must write and thank you for your able article on my letter to the Committee of the North-West London Hospital, and to inform you of the result. The Baron de Hirsch, having seen your article, sent me a cheque for £1,000, to prevent the necessity of having to close the men's ward. The immense sums expended on charity by the Baron abroad may not be known in England, but it has rendered his name famous on the Continent. And I may point out that this is not the first time he has come to the aid of the North-West London Hospital, as the Benefactors' Board shows. In consequence of your article Mr. John Maple offered £100, provided twenty other donors of £50 each could be found; and I hope these conditions may be completed. The above gifts may appear large, and they are, yet the expenditure so far outstrips the receipts, that we are nearly in a similar position as in January last, when I wrote my letter to the Committee. Again I thank you for your able advocacy on behalf of the hospital."

A fancy fair, entitled "A Dream of Health for Sick Children," was held in Kensington last week, the object of which was to raise the bulk, or, if possible, the whole, of the £700 required for furnishing the convalescent home for the Victoria Hospital. The stalls were arranged so as to represent scenes from the works of Charles Dickens, a few of whose autographs were for sale at the "Old Curiosity Shop." In addition to the usual attractions of fish-ponds and palmistry, dramatic, vocal, and artistic performances were provided by several well-known artistes who had kindly offered their services. Princess Louise, Patroness of the hospital, was to have opened the bazaar, but was unfortunately prevented.

The annual meeting of the members of the Charity Organisation Society was held on April 23, under the presidency of Mr. Timothy Holmes, the Chairman of the council. Mr. Holmes took the opportunity of explaining that the reason the majority of the Council had opposed "General" Booth's scheme was that it utterly rejected all organisation. They believed that whatever merits the plan possessed, and he was not going to deny that there were great merits in it, they would have been much enhanced and increased if General Booth had been able to submit to work with his fellow labourers. The Archbishop of Canterbury, who moved the adoption of the report, said he was convinced that this society, into who e working he had carefully examined, was doing a work of immense value now, and that it was laying the foundation of a very much finer work for all societies and all charitable persons in the future.

The festival dinner of the Royal Hospital for the Paralysed and Epileptic (Albany Memorial) was held at the Hotel Metropole on the 23rd ult., the Right Hon. the Earl of Derby presiding. The special objects of the festival were to raise a sum of £2,500, to cover the deficit on the general purposes account, and to prevent the closing of certain of the wards in consequence, and to raise a sum of £500 to complete the equipment of the new surgical wing which is now in course of construction. Every year some £8,000 has to be raised over and above the annual income, in order to meet the expenditure, though the cost per in-patient has during the last five years been gradually reduced from £78 to £67. The President said that in 1890 the hospital had a daily average of 149 patients. This represented a very important work amongst the community, and he therefore appealed with confidence to the public for aid to an excellent institution. Subscriptions to the amount of £1,870 were announced in the course of the evening.

The first annual meeting held in connection with the Lock Hospital, Westbourne Green, took place at 81, Dean Street, Soho, on the 23rd ult., Lord Kinnaird in the chair. The report presented by the Secretary, Mr. Algernon C. P. Coote, said that 731 patients had passed through the female hospital, the average cost per patient being 16s. 9d. per week, while the average stay was seven weeks. Two hundred and twenty-five in-patients had been treated at the male hospital during the same time. The total receipts in 1890 were £7,579, only £596 less than those for 1889, even though in that year the hospital received £2,200 from the festival dinner. The expenditure amounted to £8,574, as against £7,887 in 1889, or an increase of £687. The rescue work at the female hospital had progressed satisfactorily, 83 having entered during the year, while £971 had been earned by the Laundry attached to the Home, as against £820 in 1889, and £625 in 1888. At least £1,500 is required in new subscriptions in order to keep the establishment in a flourishing condition.

At the quarterly general court of the Governors, held at the Radcliffe Infirmary, Oxford, on Wednesday, a discussion took place as to the report recently published by us from "Our Special Commissioner," who had pointed out certain defects in the institution as it at present stands.

The Committee of Management presented a report on the subject, in which they characterised the "charges" as being "on the whole of a very frivolous character." Summarised briefly, the report agrees that the basement, the nurses' quarters, the interest taken by the public in the institution, and the condition of the old buildings, are not all that could be wished for. As to the condition of the walls, baths, and other appliances in the chief surgical ward, the Committee say: "The walls and ceilings are carefully cleansed, washed, and painted where necessary every year. All the baths in the ward are good. . . . The sanitary arrangements are perfectly satisfactory, the ward furniture is good, spring mattresses are almost universally employed, the lockers and tables for the patients are of a new and excellent pattern." As to the state of the Parian cement in this ward they continue: "It is not possible to give so direct a denial to the charges. . . . If Parian cement is, as The Hospital asserts, extremely absorbent, it would no doubt be better that it should be painted or periodically colour-washed; but in the tenth annual report to the Local Government Board, re-issued in 1884, the Medical Officer, Dr. Thorne Thorne, says that Parian cement well put on is well adapted to the purpose of hospital wards; and as the accident ward is quite free from those illnesses which usually arise from walls saturated 'to half the depth of the cement' with impurities, as, according to the theory of The Hospital correspondent, our walls must be, your Committee venture to question the correctness of the information of The Hospital on this point." The Committee "venture to think" that "no visitor would consider the appearance of the staircase of the Jubilee wing unworthy, neither can they discover any inadequate fitting in this ward."

The Treasurer (Dr. Bright), who presided, in moving the adoption of the report, said that the hospital had been accused of sleepiness and inactivity. However, when he mentioned that during the past seven years there had been an entire reorganisation of the nursing department, and that £1,000 had been spent on the drainage, which was as perfect as it could; that new women and children's wards had been erected; that the kitchen had been reorganised; and that the corridors had been decorated and heated so as to fit them for an ambulatory for the patients, he did not think anyone could adhere to such a charge. He gave The Hospital Commissioner credit for not being antagonistic to the hospital. He believed that he intended to point out certain things which were wanted, and by putting them in very strong language to excite people's minds, and so get the Infirmary placed in thorough good order. He could not help thinking that the impression which would remain in the mind of a person used to hospitals, after a cursory inspection, would be that the Infirmary looked sad and gloomy. Two years after he had been selected as Treasurer, plans for the reconstruction of the hospital had been submitted, and these plans were being carried out bit by bit as the Committee were able to do it. In order to fully carry out the scheme, it would be necessary to build another block for the men's wards, and then the old building could be utilised as the administrative and for the nurses' quarters. This would cost about £10,000, viz., new block £4,000, corridors connecting all blocks £1,000, and altering and bringing the old building up to date £5,000. If that could be done all that was necessary and feasible would have been done.

Canon Ashurst said that the Governors had felt that the Infirmary was not perfect, that it required amendment, and so during the past thirty years something like £30,000 had been spent in bringing it up to date.—The Rev. C. J. H. Fletcher agreed with the Committee's report, but admitted that there was a grain of truth in what The Hospital said, and thought if the Governors were wise they would give ear to it. Their London critic had put his finger on the weak point of the institution. The main building was antiquated; its three wards were unsatisfactory, and the accommodation it provided for the nurses was inadequate. The Committee did not deny the existence of these defects, as was shown by their last report. He doubted the wisdom of using the old buildings, and thought the Committee ought to consider whether it would not be better to do away with them altogether, and to erect a new front block altogether. He gave notice of the following resolution: "That the question be referred to the Committee of Management to consider, and that they shall report to the next quarterly Court what steps, if any, shall be taken for the entire reconstruction of the old parts of the Infirmary buildings."—A working-men's representative said that they, at any rate, took an active interest in the prosperity of the Infirmary.

The Rev. C. H. Daniell thought too much courtesy had been shown to our Commissioner, whom he would have treated with far less courtesy and at far less length.—Mr. A. Winkfield, on behalf of the medical staff, passing by the minor complaints as to the appearance of the buildings, thought it right to protest against the strictures as to the work and sanitary condition of the hospital. There had been an enormous improvement in the Infirmary during the thirty years he had been connected with it. He entirely agreed with the plan the Chairman had mentioned, of erecting a new men's block, and using the old buildings for the nurses' department and the management.—The report was adopted, and the proceedings terminated.

